# *In Silico* Analysis Determining the Binding Interactions of NAD(P)H: Quinone Oxidoreductase 1 and Resveratrol via Docking and Molecular Dynamic Simulations

**DOI:** 10.26650/eurjbiol.2023.1352396

**Published:** 2023-11-23

**Authors:** Santosh Kumar Behera, Christoffer Briggs Lambring, Albina Hashmi, Sriharika Gottipolu, Riyaz Basha

**Affiliations:** 1 National Institute of Pharmaceutical Education and Research, Ahmedabad, India; 2 University of North Texas Health Science Center at Fort Worth, Texas, USA; 3 University of Texas at Austion, Austin, Texas, USA; 4 The University of Texas Medical Branch, Galveston, Texas, USA

**Keywords:** Molecular Dynamic Simulation, NQO1, Resveratrol, Oxidative Stress, *In silico* Analysis

## Abstract

**Objective:**

NAD(P)H: Quinone oxidoreductase1 (NQO1) plays a crucial role in cellular defense against oxidative stress. Overexpression of NQO1 is linked to various cancer pathways. Despite its potential, the actual mechanisms to inhibit NQO1 and increase the efficacy of standard therapeutic options are not yet established. Resveratrol is an anti-cancer polyphenol found in dietary products and red wine. The objective of this investigation is to employ in silico methods to explore how resveratrol interacts with NQO1.

**Materials and Methods:**

Docking analysis of resveratrol against NQO1 was performed using Glide. The most efficiently docked complex was characterized and analyzed by measuring intermolecular (IM) hydrogen (H)-bonds and binding energy values, additional hydrophobic, and electrostatic interactions. IM interaction between complexed protein and compound was demonstrated using LigPlot+ and the Schrödinger ligand interaction module. Molecular dynamics tools were employed to examine the physical movement of molecules to evaluate how macromolecular structures relate to their functions.

**Results:**

The results of this investigation depicted a strong affinity of resveratrol against NQO1 followed by MD simulations (NQO1-resveratrol complex-binding energy: −2.847kcal/mol). Resveratrol’s robust binding affinity through docking and molecular dynamic simulations highlights a significant change around 90 ns. The H-bonds number was inversely linked with the resveratrol-NQO1 complex stability. The NQO1-Resveratrol complex displayed dynamic motion, as revealed by porcupine projections, indicating alterations in its movement and flexibility.

**Conclusion:**

The present *in silico* analysis suggests a possible alteration in resveratrol’s orientation in the protein binding pocket. The findings encourage further investigation, including validation using *in vitro* and *in vivo* assays.

## INTRODUCTION

NAD(P)H: Quinone oxidoreductase 1 (NQO1) is a multifunctional enzyme encoded by the NQO1 gene. It functions as an effective cytoprotective agent, a protective antioxidant, and a regulator of the oxidative stressors that cause DNA damage in cancer cells in chromatin-binding proteins.^1–3^ Upregulation of NQO1 is observed in numerous human cancers.^4–8^ It is established that the elevation of NQO1 levels has been attributed to the cellular defense response against increased oxidative stress associated with cancer. NQO1’s induction is driven by transcriptional activation through the Keap1/Nrf2 pathway, which is frequently dysregulated in cancer cells.^9–12^ The heightened NQO1 levels in cancer cells confer a survival advantage by enabling better oxidative stress management, facilitating tumor growth, and potentially contributing to treatment resistance.^13–15^

Understanding the intricate relationship between cellular oxidative stress, redox balance, and NQO1’s participation in cellular responses holds significant implications for multiple fields.^3,16^ Targeting NQO1 and the related pathways could offer novel strategies for therapeutic interventions in cancer treatment, exploiting the dependency of cancer cells on redox adaptation. Multiple iterations of NQO1 regulating or bioactivating methods have already been explored for cancer therapy and diagnostic efforts.^17–19^ Further investigations are warranted to decipher the complex interplay between NQO1, ROS generation, and its multifaceted roles in cellular stress responses and carcinogenesis.

Resveratrol, a polyphenol belonging to the stilbenoids family, has two phenol rings joined by an ethylene bridge. Resveratrol (3,5,4-trihydroxy-trans-stilbene) is a polyphenol discovered in dozens of plant species, including the skin and seeds of grapes,^20–22^ red wines, and various human diets. Resveratrol exerts its anti-cancer effects through multiple mechanisms. Not only does resveratrol display antioxidant properties, as mentioned above, but it also exhibits more pleiotropic effects, including direct anti-tumor activity. Acting on pathways like Wnt/β-catenin, TGF-β/SMAD, and PI3K/Akt/mTOR, resveratrol can inhibit multiple pathways of tumor progression and metastasis.^23–26^

In this investigation, an *in silico* approach is performed to study the anti-cancerous activity of resveratrol through its inhibitory potential against the NQO1 protein. The *in silico* docking approach depicted a better binding affinity of resveratrol against NQO1, followed by molecular dynamics (MD) simulations, which tracked the trajectory graphs, with a sudden increase in their peaks, particularly at the 90-nanosecond (ns) mark of the MD simulation time period, which is a matter of interest in this investigation. The sudden rise in the peaks may be due to a sudden change in the orientation of resveratrol in the binding pocket of the protein. Therefore, based on the findings, we recommend that the molecule be synthesized and *in vitro* and *in vivo* analyses conducted to corroborate the efficacy seen *in silico* to gauge their potency as anti-cancerous drugs before clinical research.

## MATERIALS AND METHODS

### *In silico* Analysis

The information on the 1) structure, 2) sequence, and 3) function of NQO1 was retrieved from the UniProtKB database with ID P15559 (NQO1_HUMAN), Protein Data Bank (PDB) Research Collaboratory for Structural Bioinformatics (RCSB). PDB ID: 1KBQ with resolution 1.80 Å was used in this study. Chain A of NQO1 was found to have 272(2-273) amino acids (aa). Other chains and co-crystallized molecules were evaluated (BIOVIA Discovery Studio 4.5).

### Prediction of Binding Site

The active site residues part of the binding site were used to predict the binding site of NQO1 following the published model.^27^

### Retrieval of Resveratrol

Resveratrol’s structural data was extracted in Structure Data Format (SDF) using the Compound ID: 445154 from the PubChem database.^28^ The structure was converted to .pdb format using BIOVIA Discovery Studio 4.5 Visualizer (BIOVIA, San Diego, CA, USA), to use in docking tools.

### Molecular Docking

Resveratrol was docked against NQO1 in extra precision (XP) mode using Glide (Grid-based Ligand Docking with Energetics), according to binding energy, IM H-bonds, and hydrophobic and electrostatic interactions, before the most viable docked complexes were analyzed further. Schrödinger’s ligand interactions module and LigPlot+ (https://www.ebi.ac.uk/thorntonsrv/software/LigPlus/) were utilized to reveal the IM links between the protein-compound complexes.

### Molecular Dynamics Simulations

As shown earlier, MD was used to assess the atom and molecule’s physical movements.^29^ For a set amount of time, the molecules and atoms interact, displaying the system’s dynamic “evolution.”^30^ Drug binding modalities were confirmed through a comprehensive view of the NQO1-resveratrol complex by performing MD simulations of the Apo(NQO1:only protein) and Holo state: NQO1-resveratrol complex using the Desmond program. The top-scoring ligand-protein complexes were analyzed by 100 nanoseconds (ns) MD simulation.

The MD process encompassed several steps: “minimization, heating, equilibration, and run generation.”^31^ Minimization of protein-ligand complexes utilized the OPLS4 force field, automatically determining topology and atomic coordinates. The ligand was placed within a 15X15X10 orthorhombic box using the SPC solvent model. To achieve physiological pH, neutralization necessitated a concentration of 0.15 M. The Particle Mesh Ewald (PME) boundary condition was employed to establish the water box, ensuring that the solute atoms remained at least 10 Å away from the box’s edges. Minimization of the protein-ligand complexes was done with the OPLS4 force field before the topology and atomic coordinates were automatically determined.^32^ The ligand was submerged in an orthorhombic 15X15X10 SPC solvent model box. The physiological pH neutralization required 0.15 M. The water box was set up via the PME boundary condition to assure no solute atoms occurred within 10 Å of the border.

Employing the NPT ensemble, the entire system underwent a simulation at 300 K for a duration of 100 ns. Subsequently, graphs depicting the root mean square deviation (RMSD) and root mean square fluctuation (RMSF) were generated. RMSD is used to measure the difference from initial structure conformation compared to its final position. The individual residue flexibility of a protein or complex can be determined by calculating the RMSF.^33^ The most likely ligand binding mode at the protein’s binding site is demonstrated in the simulated interaction diagram.^34^

### Principal Component Analysis (PCA)

By reducing high-dimensional motional sets of data into a manageable subset made up of principal components (PCs) that characterize the collective motion, a PCA or Essential Dynamics (ED) separates collective motions from local dynamics.^35^ The ED approach was used to run PCA utilizing the Desmond module of Schrödinger Maestro v 2022.4 to achieve the motions in the Apo and Holo states.

## RESULTS

### Analysis of Binding Sites and Grid Scores of Targeted Protein NQO1

The consensus results from each web server represented the residues. Active site formation of NQO1 involves the following amino acids: His11, Ser16, Thr15, Phe17, Asn18, Ala20, Pro102, Trp105, Phe106, Leu103, Thr148, Thr147, Gly149, Gly150, Tyr155, Ile192, Arg200, and Leu204. To screen compounds against potential targets, a well-known docking software system called AutoDock tool (ADT) was used.^27^ ADT v.1.5 was used to assign Kollman charges to the protein. The dimensions, spacing, and parameters used to build the NQO1 grid were chosen to help the ligand/drug’s fully extended conformation. The centering values for the x, y, and z axes were 22.072, 12.323, and 13.297, respectively.

### Molecular Docking

**Figure 1.**
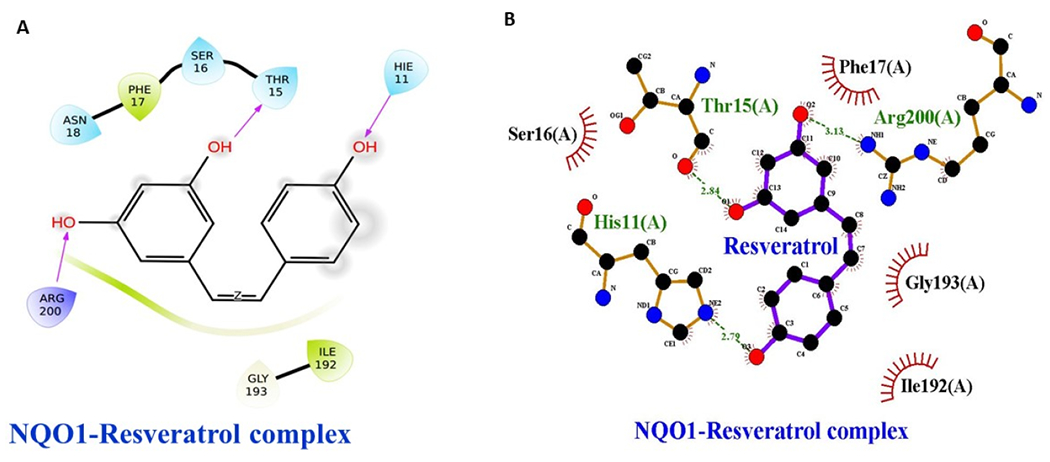
Binding energies and other interaction studies of the NQO1-resveratrol complex. (A) The NQO1-resveratrol complex exhibited IM hydrogen bonding, electrostatic interactions, and hydrophobic interactions. The 2D representation was generated using the ligand interactions module of Schrödinger. (B) The NQO1-resveratrol complex displayed its 2D interaction pattern through the utilization of the LigPlot+ tool and BIOVIA Discovery Studio 4.5 Visualizer (BIOVIA, San Diego, CA, USA).

The binding energies and other interaction studies of the the NQO1-resveratrol complex (Table 1; Figures 1A and 1B) showed that the drug-target interactions binding energies varied. There were several conformations produced from the docking research analysis utilizing GLIDE, but only the most favorable configuration with the maximum docking score was carefully selected for the IM interaction investigation. The results reflected that −2.847 kcal/mol was the binding energy for the NQO1-resveratrol complex. To comprehend and check the binding modalities of “protein-ligand interaction” for a certain time period, MD simulations of the docked complex were performed.

### Trajectory Analysis of MD Simulations

**Figure 2.**
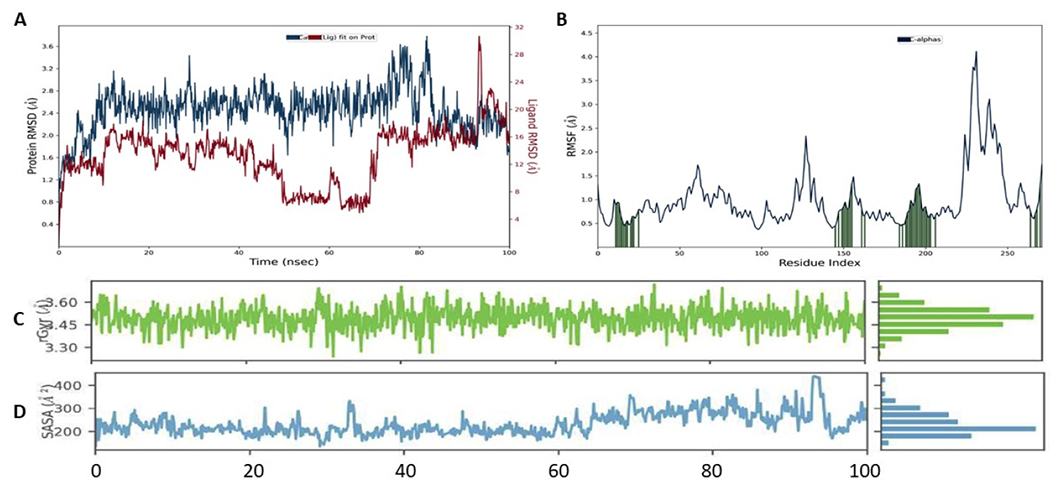
Root mean square deviation of the NQO1-resveratrol complex. The conformational stability of the NQO1 protein’s Apo and Holo states was assessed over a 100 nanoseconds (ns) duration of molecular dynamics simulation using the following analyses: (A) Backbone-RMSD of the NQO1-resveratrol complex. (B) Cα-RMSF profile of the NQO1-resveratrol complex. (C) Radius of gyration (Rg) profile of the NQO1-resveratrol complex. (D) Solvent accessible surface analysis (SASA) of the NQO1-resveratrol complex.

MD was used to analyze atom/molecule physical movements, as described above.^30^ A 100 ns MD simulation was used to test the stability of the docked complex with compound and receptor structural rearrangements. In order to comprehend the dynamic behavior and mode of binding, the dynamics and stability of two systems (NQO1: Apo; NQO1-resveratrol complex: Holo) were assessed using the Desmond suite (Schrödinger Release 2022-4: Maestro, Schrödinger, LLC, New York, NY, 2022). The dynamic stability of both systems (Apo and Holo) was assessed using the RMSD profile of the backbone atoms at 100 ns (Figure 2A). After 75 ns of MD simulations, the backbone RMSD graph of the Holo state showed a stable trajectory when compared to the Apo state.

Throughout the MD simulation run, Apo displayed aberrations compared to its Holo condition. In contrast to the Apo state, which showed significant variations over the course of the MD simulations, the Holo state displayed a stable RMSD value between ~1.6 and ~2.8 for the 75 to 100 ns of simulation time. This illustrates how protein can be stabilized by reversing the effects of resveratrol. The RMSD result was further validated by the variation of residues using RMSF. An RMSF graph (Figure 2B) was used to track the movement of specific residues in both states. This could be because resveratrol interaction affected the amino acid residues between 60 and 70, 125 and 130, and 220 and 240, all showing higher changes in their Cα atoms than other sites. The terminal residues, approximately 10 in number, displayed greater fluctuations at both their C- and N-terminal ends across all states, but these variations can be considered negligible. Residues within the protein that engage with the ligand are marked with vertical green bars.

The interactions among amino acids are influenced by their exposure to specific solvents, particularly through hydrophobic interactions. The degree of exposed surface area is inversely correlated with the frequency of these interactions with the solvent and critical protein residues. A decrease in the solvent surface that was accessible in the holo state has been depicted in the SASA graph (Figure 2D).

### H-Bond Analysis

**Figure 3.**
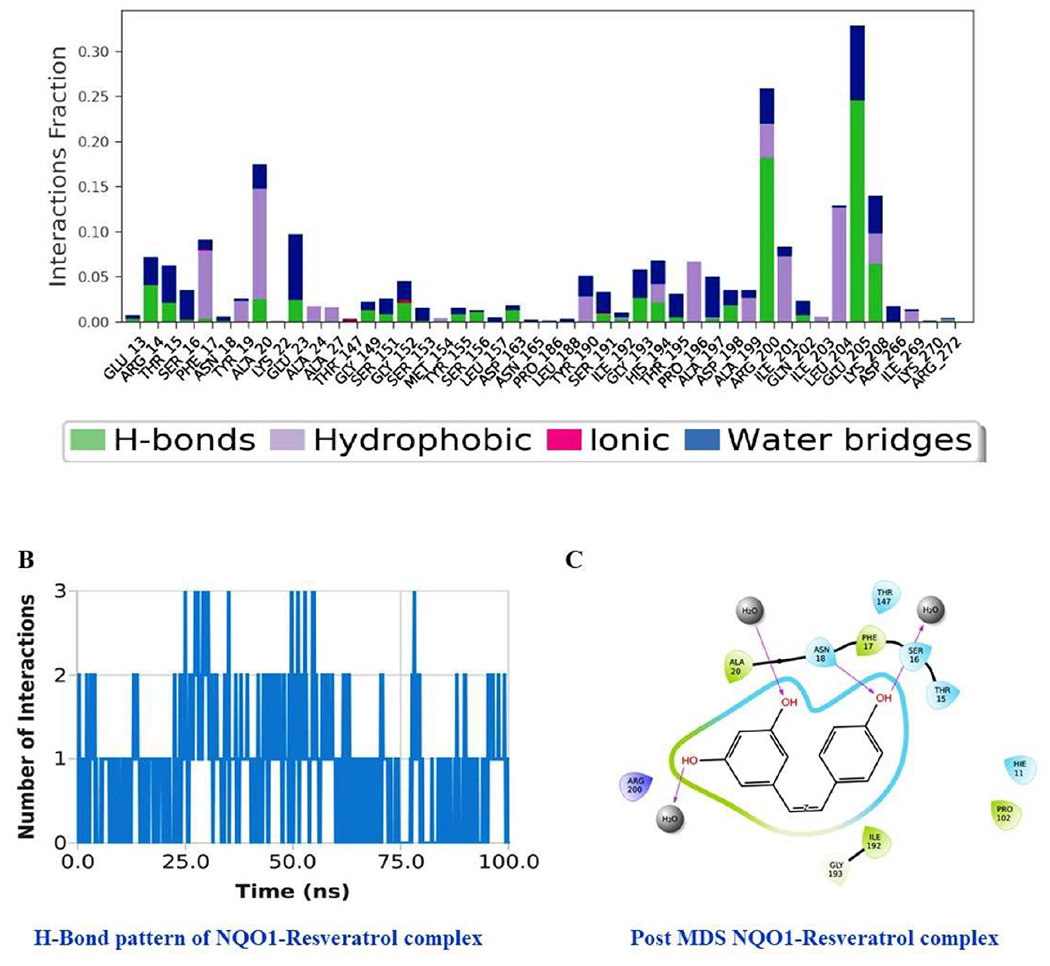
H-bond analysis of the NQO1-resveratrol complex. (A) The protein-ligand contacts within the NQO1-resveratrol complex throughout the 100 ns simulation are visualized through a stacked bar chart. (B) The fluctuations in hydrogen bond interactions within the NQO1-resveratrol complex during the 100 ns simulation are indicated by blue lines. (C) Following the MD simulations, interactions including IM hydrogen bonding, electrostatic, and hydrophobic contacts are depicted within the NQO1-resveratrol complex. This graphical representation was generated using the ligand interaction module of Schrödinger.

Schrödinger Release 2022-4 was used to visualize the IM hydrogen bonds of the Holo state during the MD simulations (Figure 3A to 3C). Variable IM hydrogen bonds were discovered during the modeling of the Holo state. In the case of the Holo state, the post-MD simulation study did not find any H-bonds. The simulation showed that the number of H-bonds was inversely linked with the stability of the resveratrol-NQO1 complex. The Holo state’s IM hydrogen bonding was observed. According to the stacked bar chart of Holo in Figure 3A, the amino acid residues Ala20, Arg200, and Glu205 of NQO1 may be necessary for the binding and control of the protein. These residues could be the most crucial amino acid residues for binding and protein function. Values exceeding 0.5 in this histogram are achievable due to some protein residues’ capacity to produce multiple interactions of the same subtype with resveratrol. The amount of IM hydrogen bonds was consistently reflected in the simulation of the Holo state (Figure 3B). In the case of the post-MD of Holo, no H-bond was visible (Figure 3C). The H-bond-forming residues of His11, Arg200, and Thr15 broke down during the simulations of Holo but were later made up for by novel hydrophobic interactions and van der Waals interactions.

### Principal Component Analysis (PCA)

**Figure 4.**
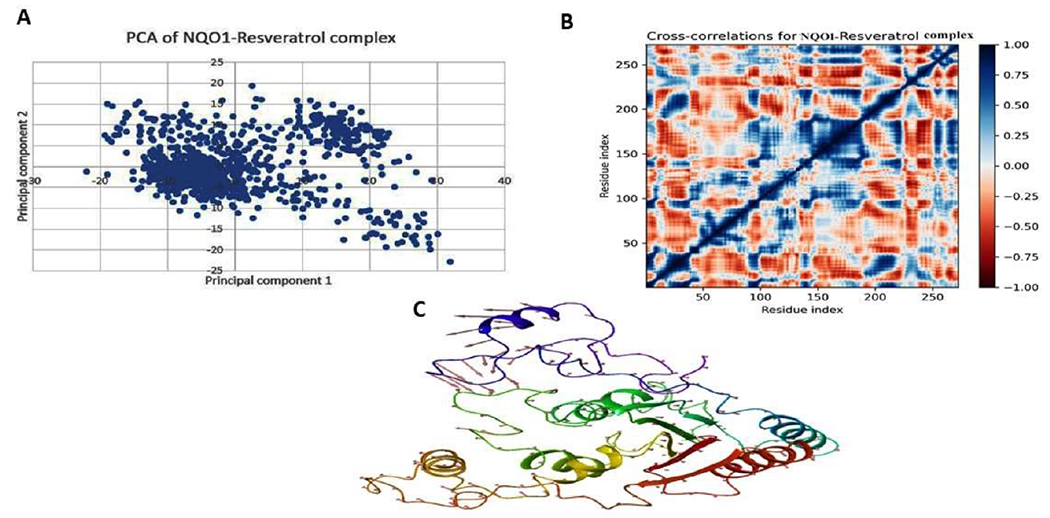
Principal component analysis of the NQO1-resveratrol complex. (A) The projection of trajectories (PC1 and PC2) is symbolized by the cloud. (B) A comparative analysis of cross-correlation matrices for the backbone atoms within the NQO1-resveratrol complex was conducted using PCA. (C) The individual components within the NQO1-resveratrol complex are visually represented through sharp porcupine plot curves in a vectorial manner.

The trace values of the covariance matrix for the backbone atoms were instrumental in limiting and characterizing the flexibility of both the apo and Holo states in each simulation protocol. The projections of trajectories along PC1 and PC2 visually depicted how these states moved within the phase space. These trajectories were mapped onto the first two principal components, providing a clear representation of the motion exhibited by the apo and Holo states of the protein-ligand complex (Figure 4A).

Higher flexibility in the Holo: NQO1-resveratrol complex is represented by the scattering cloud of PCA plots. The atom configurations may have moved and moved back during the dual time course of the 100 ns simulation time frame, which could account for the flexibility. The “Cross-correlation matrix” of the Cα- displacement revealed that all of the residues in the “NQO1” protein had motions that were both negatively (Figure 4B, blue shade) and positively (Figure 4B, red shade) linked with them, supporting the protein’s erratic movement.

The vectorial representation of its individual components depicted the direction of motion. The majority of internal and external motions were visible in the projection vectors. Sharp porcupine curves were noticed after the graphing (Figure 4C). The NQO1-resveratrol complex was shown by the porcupine projection as inward and outward motion, which signify changes in motion and flexibility. This may result from atom configurations moving and then moving back throughout the 100 ns simulation time frame.

Oxidative stress, originating from the presence of disproportionate reactive oxygen species (ROS) generation and cellular antioxidant defenses, can be mitigated by NQO1’s enzymatic activities. Recent studies have highlighted the potential of NQO1 inhibitors to impact cellular responses, particularly in the context of apoptosis induction.

## DISCUSSION

The NQO1-resveratrol complex’s interaction pattern involving IM hydrogen bonding, electrostatic interactions, and hydrophobic interactions is a crucial aspect of understanding the molecular basis of their binding and potential biological activity. These results have been presented in Table 1 and Figure 1. Hydrogen bonds are critical to stabilize molecular complexes. In the tested NQO1-resveratrol complex, the IM hydrogen bonds form between specific atoms on both molecules, which potentially contribute to the overall stability of the complex.^36,37^ Analyzing hydrogen bonding patterns can reveal key binding interactions and help explain the complex’s biological activity.^38,39^ The electrostatic interactions result from the attraction or repulsion of charged particles, such as positively charged (cationic) and negatively charged (anionic) groups on molecules. In the context of the NQO1-resveratrol complex, electrostatic interactions might involve charged regions on both molecules that come into close proximity during binding. Understanding these interactions is essential for elucidating the electrostatic contributions to the complex’s stability and function.^29,40^ Hydrophobic interactions can occur between nonpolar or hydrophobic regions of molecules, with such interactions possibly contributing to stabilizing protein-ligand complexes.^41^ Resveratrol, being a polyphenolic compound with a hydrophobic core,^42^ is likely to engage in hydrophobic interactions with hydrophobic patches on NQO1 or other nearby residues. These interactions can facilitate the binding of resveratrol to NQO1 and influence its biological effects. Schrödinger’s Maestro and the Desmond v 2022.4 software suite are commonly used for molecular modeling and simulation studies. In the context of the NQO1-resveratrol complex, it likely offers advanced visualization and analysis capabilities, enabling researchers to produce detailed 2D interaction diagrams that visually represent the specific interactions between NQO1 and resveratrol. This aids in a more comprehensive understanding of the complex’s binding pattern.^30,43^ Overall, the description of the NQO1-resveratrol complex’s interactions and the use of these computational tools reflect a systematic and in-depth approach to studying molecular interactions.^44–46^ This knowledge is essential for designing and optimizing drug candidates, understanding biological mechanisms, and potentially developing new therapeutics or interventions based on the NQO1-resveratrol interaction.

The assessment of both the stability of the docked complex involving the compound and any structural rearrangements in the receptor was conducted through MD simulations (Figure 2).

The fluctuation in resveratrol’s rGyr within the protein’s receptor binding pocket remained consistent, spanning approximately ~3.40 Å to ~3.70 Å. This consistent ligand behavior was evident throughout the 100 ns MD simulation, indicating stability. Notably, this illustrates that the Holo state maintains greater compactness, underscoring the inverse correlation between rGyr values and compactness.^35^ The RMSF analysis lends robust validation to the rGyr findings. Hydrophobic interactions influence how amino acids interact with solvents by modulating their exposure. The more an amino acid is exposed to a solvent, the less frequent its interactions with that solvent, with crucial protein residues following this pattern. Figure 2D in the SASA graph illustrates a decrease in solvent-accessible surface area in the Holo state, reflecting this effect. The SASA analysis revealed that resveratrol’s binding induced changes in the hydrophilic and hydrophobic interaction regions. This phenomenon could potentially lead to alterations in protein surface orientations, driven by the relocation of amino acid residues from accessible to buried areas. Over the course of a 100 ns MD simulation, the SASA graphs for the Holo state depicted SASA values spanning approximately ~200 to ~360 Å. This suggests that the protein surface orientation may change due to the amino acid residue moving from the accessible area to the buried area. Taking together all the trajectory graphs, a sudden increase in the peak, particularly at 90 ns MD simulation time period, could be observed, which is a matter of interest in this investigation. The rise in the peak may be due to the sudden change in the orientation of resveratrol in the binding pocket of the protein.^47^

In the study using Schrödinger Release 2022-4, MD simulations were employed to investigate the behavior of IM hydrogen bonds in the Holo state of the resveratrol-NQO1 complex (Figure 3A to 3C). Variable IM hydrogen bonds were observed during the modeling of the Holo state, but interestingly, no hydrogen bonds were found in the post-MD simulation of the Holo state. This suggests that the stability of the resveratrol-NQO1 complex was inversely related to the presence of hydrogen bonds. The stacked bar chart for the Holo state (Figure 3A) highlights specific amino acid residues, namely Ala20, Arg200, and Glu205 of NQO1, which may play a crucial role in the binding and regulation of the protein. These residues appear to be vital for both binding and the protein’s overall function. The histogram indicates that values exceeding 0.5 are possible, indicating that specific protein residues have the capacity to form multiple interactions of the same type with resveratrol. This pattern of hydrogen bond behavior was consistently observed throughout the simulation of the Holo state (Figure 3B). However, in the post-MD analysis of Holo, no hydrogen bonds were evident (Figure 3C). During the Holo simulations, specific hydrogen bond-forming residues like His11, Arg200, and Thr15 initially broke their hydrogen bonds but were subsequently compensated by new hydrophobic interactions and van der Waals interactions. This suggests a dynamic and adaptive behavior of the complex during the simulation, where hydrogen bonds were replaced by other types of interactions. Overall, these findings provide insights into the role of hydrogen bonds and other interactions in the stability and dynamics of the resveratrol-NQO1 complex, with specific amino acid residues like Ala20, Arg200, and Glu205 appearing to be critical players in the binding and function of the protein.

The covariance matrix trace values of backbone atoms were critical in governing and defining the flexibility of the apo and Holo states in each simulation protocol. These values provide insights into how atoms within the protein-ligand complex move and interact. Trajectory projections based on PC1 and PC2 illustrated the dynamic behavior of the states within the phase space. This type of analysis helps visualize how the protein and ligand move and evolve during the simulation. In Figure 4A, PC1 and PC2 were used to project the trajectories of the apo and Holo states of the protein-ligand complex. The scattering cloud in the PCA plots for the Holo state suggests higher flexibility in the NQO1-resveratrol complex. This variability in atom configurations may involve movements that occur and then reverse over the course of the 100 ns simulation, contributing to the overall flexibility observed.

The “Cross-correlation matrix” analysis of C-displacement (Figure 4B) reveals that all residues in the NQO1 protein exhibit both positively (red shade) and negatively (blue shade) correlated motions. This indicates that the protein undergoes complex, coordinated movements during the simulation, with some residues moving in the same direction, while others move in the opposite direction. The vectorial representation of individual components (porcupine plots, Figure 4C) indicates the direction of motion. These plots illustrate both internal and external motions, with sharp curves in the porcupine plots suggesting significant changes in motion and flexibility. In the case of the NQO1-resveratrol complex, the porcupine projection indicates both inward and outward motions, suggesting that atom configurations are not only changing but also returning to previous states during the 100 ns simulation time frame.

Overall, these analyses provide valuable insights into the dynamic behavior of the NQO1-resveratrol complex, highlighting the complex interplay of motions, flexibility, and structural changes that occur during the simulation. These findings can aid in understanding the conformational dynamics of the protein-ligand complex and its functional implications.

## CONCLUSION

Oxidative stress, originating from the presence of disproportionate ROS generation and cellular antioxidant defenses, can be mitigated by NQO1’s enzymatic activities. Recent studies have highlighted the potential of NQO1 inhibitors to impact cellular responses, particularly in the context of apoptosis induction. Understanding the intricate relationship between cellular oxidative stress, redox balance, and NQO1’s participation in cellular responses holds significant implications for multiple fields. Targeting NQO1 and related pathways could offer novel strategies for therapeutic interventions in cancer treatment, exploiting the dependency of cancer cells on redox adaptation. Further investigations are warranted to decipher the complex interplay between NQO1, ROS generation, and its multifaceted roles in cellular stress responses and carcinogenesis.

**Figure 5.**
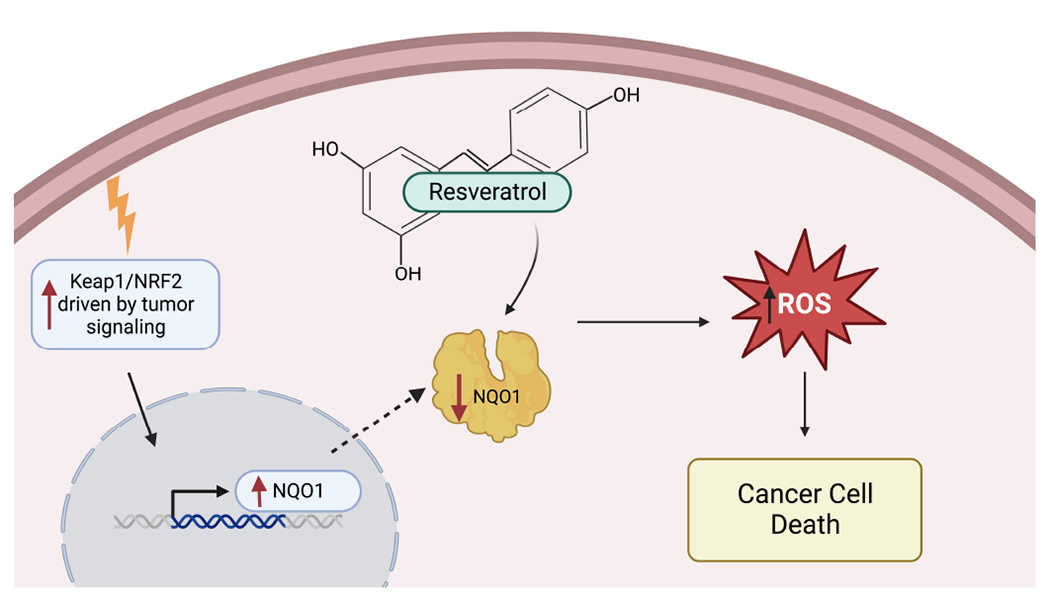
Schematic representation of the mechanism of resveratrol interaction with NQO1 and its implication as a potential cancer therapy. Upon dysregulation of Keap1/NRF2 pathways driven by pro-tumorigenic signaling in cancer cells, NQO1 transcription and translation are increased. We suggest upon binding with resveratrol, NQO1 is downregulated, leading to an elevated level of intracellular ROS resulting in increased cancer cell death.

Cellular oxidative stress, stemming from disruptions in redox equilibrium, is intimately linked to ROS generation, prominently through enzymatic activities involving NQO1. This cytosolic reductase is integral to cellular stress responses and is notably upregulated in various human cancers. Elucidating NQO1’s precise functions within the context of oxidative stress and cancer has the potential to unravel novel avenues for therapeutic innovations and deepen our understanding of cellular adaptation to stress conditions. When the Keap1/NRF2 pathways, influenced by cancer-promoting signals, become disrupted in cancer cells, there is a surge in the transcription and translation of NQO1. Resveratrol may bind to NQO1, reduce its activity, and subsequently raise the levels of intracellular ROS.^48^ This process ultimately leads to heightened cancer cell mortality (Figure 5).

This investigation utilizing the *in silico* docking approach depicted a strong binding affinity of resveratrol against NQO1. Notably, following the trajectory graphs through MD simulations revealed a sudden increase in the peaks particularly at 90ns MD simulation time period. The rise in the peak may be due to the sudden change in the orientation of resveratrol in the binding pocket of the protein. In addition, the trajectory analysis suggested resveratrol’s potential to destabilize protein structure, corroborated by global and local changes in the RMSD and RMSF readings and SASA data. The RMSF values supported the rGyr findings wherein the Holo state was more compact, and multiple residues were identified as potential keys for resveratrol-NQO1 binding interactions. These findings warrant the need for subsequent preclinical studies to verify presented *in silico* results, potentially leading to clinical research involving resveratrol and NQO1 interactions.

## Figures and Tables

**Table 1.** Molecular docking scores of resveratrol against human NQO1.

SI. No.	Target	PubChem CID	Drug	Binding Energy(kcal/Mol)	No. of H-Bonds	H-Bond Forming Residues	Average Distance of H-Bonds (Å)
**1.**	NAD (P) H dehydrogenase [quinone] 1 (**NQO1**)	445154	Resveratrol	−2.847	3	His11, Arg200, Thr15	~2.045
